# Unexpected guests in the tumor microenvironment: microbiome in cancer

**DOI:** 10.1007/s13238-020-00813-8

**Published:** 2020-12-09

**Authors:** Abigail Wong-Rolle, Haohan Karen Wei, Chen Zhao, Chengcheng Jin

**Affiliations:** 1 Thoracic and Gastrointestinal Malignancies Branch, National Cancer Institute, National Institutes of Health, 4 Memorial Drive, Bethesda, MD 20892 USA; 2 Department of Cancer Biology, Perelman School of Medicine, University of Pennsylvania, 421 Curie Blvd., Philadelphia, PA 19104 USA

**Keywords:** microbiome, tumor, lung cancer, immune system, tumor-associated microbiota, cancer immunotherapy

## Abstract

Although intestinal microbiome have been established as an important biomarker and regulator of cancer development and therapeutic response, less is known about the role of microbiome at other body sites in cancer. Emerging evidence has revealed that the local microbiota make up an important part of the tumor microenvironment across many types of cancer, especially in cancers arising from mucosal sites, including the lung, skin and gastrointestinal tract. The populations of bacteria that reside specifically within tumors have been found to be tumor-type specific, and mechanistic studies have demonstrated that tumor-associated microbiota may directly regulate cancer initiation, progression and responses to chemo- or immuno-therapies. This review aims to provide a comprehensive review of the important literature on the microbiota in the cancerous tissue, and their function and mechanism of action in cancer development and treatment.

## INTRODUCTION

The commensal microbiome contains at least 100-fold more unique genes than the human host genome (Ley et al., 2006; Human Microbiome Project, 2012; Belkaid and Hand, 2014; Dzutsev et al., 2017). The primary habitat of the human commensal microbiota is the gut, but thriving microbial populations exist throughout much of the body including the skin, oral, respiratory, and genital tracts (Human Microbiome Project, 2012). These microbes influence many of our important physiological functions including our metabolism and immune system. This metaorganism, made up of human host and microbial symbionts, senses environmental cues and adapts accordingly (Belkaid and Naik, 2013; Dzutsev et al., 2017). The complex interaction between the host and microbes extends to cancer; microorganisms are implicated in 20% of human malignancies (de Martel et al., 2012). Commensal bacteria directly affect tumorigenesis, progression and responses to treatment. Disruption of commensal gut microbiota in mice can affect the response of tumors to immunotherapy and chemotherapy (Iida et al., 2013; Viaud et al., 2013; Sivan et al., 2015; Vétizou et al., 2015). This dependence of treatment responsiveness on the gut microbiome has also been observed in cancer patients. Antibiotic treatment is associated with reduced response to immune checkpoint inhibitors whereas the presence or increased abundance of some gut bacteria strains correlates with better outcomes (Gopalakrishnan et al., 2018; Matson et al., 2018; Routy et al., 2018). The systemic effect of the gut microbiome in regulating cancers in the gastrointestinal tract, as well as in distal sites, has been well reviewed previously (Garrett, 2015; Dzutsev et al., 2017; Shalapour and Karin, 2020). The focus of this review is the role of microbiota in the tumor-bearing tissue, especially intratumor microbes.

The distribution of commensal microbes across anatomical sites reveals distinct microbial communities (Costello et al., 2009; Belkaid and Hand, 2014). The commensal microbiota of tumor-bearing tissue makes up an important part of the tumor microenvironment, affecting tumorigenesis and tumor progression on a more local scale (Garrett, 2015). Additionally, the populations of bacteria that reside specifically within tumors have been found to be tumor-type specific, suggesting an association with tumor development (Nejman et al., 2020). In a very recent study, Nejman et al. profiled over 1500 tumors across seven cancer types and discovered that intratumor and intracellular microbial compositions are distinct between tumor types; further analysis revealed correlations between microbial metabolic pathways and clinical features (Nejman et al., 2020). The distinction between the role of commensal microbes in tumor-bearing tissue and the role of intratumor or tumor-associated microbes has not always been made and cannot always be readily distinguished due to technical challenges. This review aims to provide an overview of the microbiota of the cancerous tissue, and their role in tumorigenesis and tumor progression across different cancer types (Table 1).

**Table 1:** **An overview of the microbiota in the cancerous tissue, and their role in tumorigenesis and tumor progression across different cancer types.**

**Cancer type**	**Name of the identified tumor-associated microbiome**	**Proposed mechanism**
Lung cancer	*Veillonella* (Yan et al., 2015; Lee et al., 2016; Tsay et al., 2018)	N/A
	*Streptococcus* (Cameron et al., 2017; Tsay et al., 2018)	N/A
	*Acinetobacter* (Cameron et al., 2017; Gomes et al., 2019)	N/A
	*Capnocytophaga* (Liu et al., 2018)	N/A
	*Thermus* and *Legionella* (Yu et al., 2016)	N/A
	*Megasphaera* (Lee et al., 2016)	N/A
	*Granulicatella adiacens*, *Enterococcus*, and *Escherichia coli* (Cameron et al., 2017)	N/A
	*Brevundimonas*, *Propionibacterium*, and *Enterobacter* (Gomes et al., 2019)	N/A
	*Prevotella* and *Rothia* (Tsay et al., 2018)	N/A
	*Acidovorax* (Greathouse et al., 2018)	N/A
Colorectal cancer	*Fusobacterium nucleatum* (Castellarin et al., 2012; Kostic et al., 2012, 2013; Rubinstein et al., 2013; Bullman et al., 2017; Yu et al., 2017; Garrett, 2019)	Binding of *F*. *nucleatum* adhesin molecule to the cell surface motifs on cancer cells or immune cells, which leads to the downstream oncogenic or immunosuppressive signaling (Garrett, 2019).
	Enterotoxigenic *Bacteroides fragilis* (ETBF) (Dejea et al., 2018)	Tumor-coating ETBF recruits other bacteria as well as immune cells to the tumor site and boosts IL-17-mediated inflammation (Dejea et al., 2018).
	*Escherichia coli* expressing the genomic island polyketide synthase (pks+ *E. coli*) (Arthur et al., 2012; Dejea et al., 2018)	pks+ *E. coli*-derived colibactin alkylates DNA and produces DNA adducts, resulting in DNA damage in colonic epithelial cells (Wilson et al., 2019).
	*Bifidobacterium* (Shi et al., 2020)	N/A
Pancreatic ductal adenocarcinoma	*Proteobacteria* (Geller et al., 2017; Pushalkar et al., 2018)	*Proteobacteria* lead to T cell anergy in a Toll-like receptor-dependent manner, accelerating tumor progression (Pushalkar et al., 2018).
	*Malassezia globose* (Aykut et al., 2019)	Contributes to tumorigenesis, tumor growth, and gemcitabine resistance via mannose-binding lectin-C3 axis (Aykut et al., 2019).
	*Pseudoxanthomonas*, *Saccharopolyspora*, and *Streptomyces* spp. (Riquelme et al., 2019)	N/A
Esophageal cancer	*Fusobacterium nucleatum* (Yamamura et al., 2016)	Contributes to tumor infiltration of Treg lymphocytes in a chemokine (especially CCL20)-dependent fashion, promoting aggressive tumor behaviors (Yamamura et al., 2016).

## MECHANISMS

**Figure 1:**
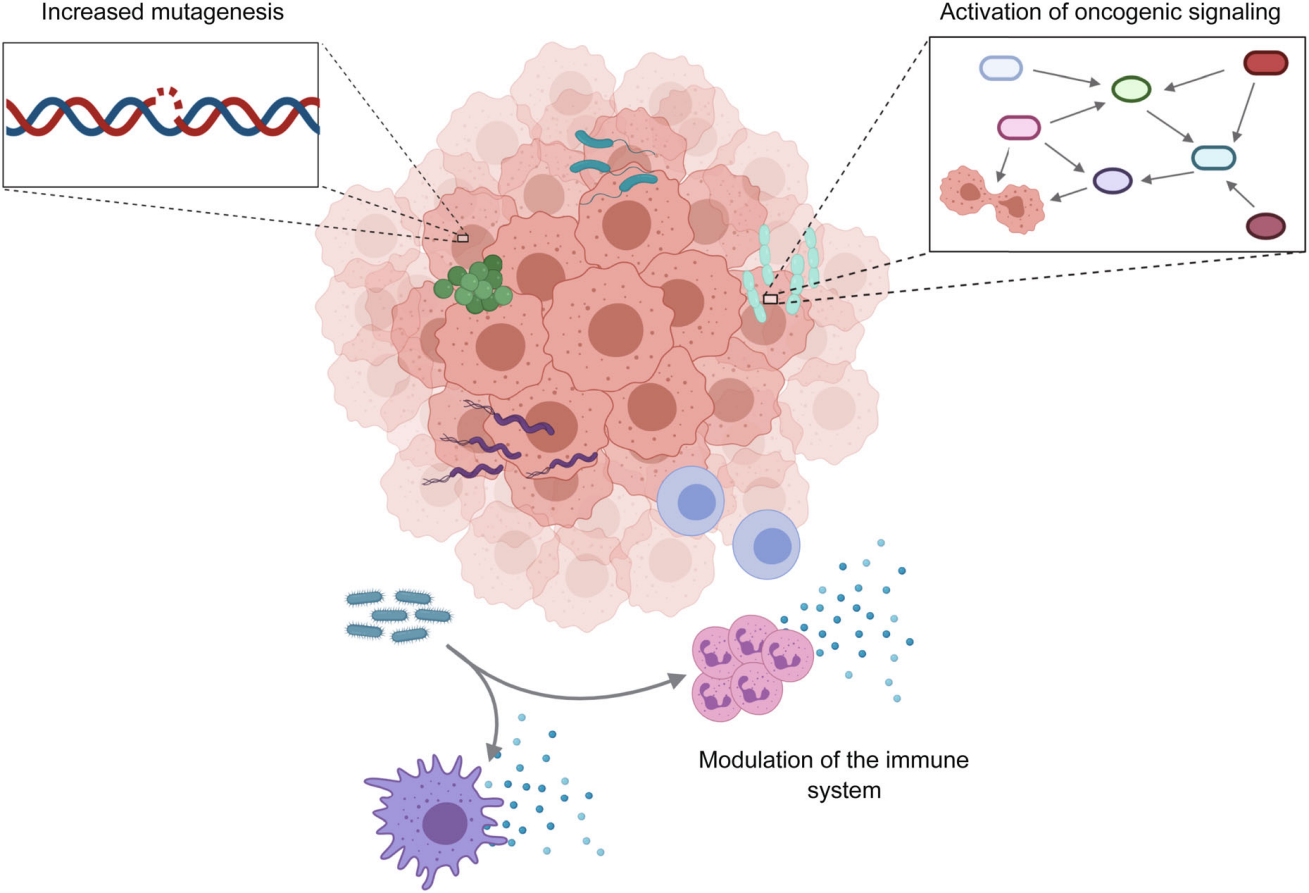
**Interactions between microbes and tumor cells.** Microbial communities in the tumor-bearing tissue and intratumor microbes are associated with cancer, but their exact role is not fully understood. Three main mechanisms have been proposed to explain how local microbes affect carcinogenesis and cancer progression: direct facilitation of DNA damage and increased mutagenesis (top left), activation of oncogenic signaling pathways (top right), and reduction or enhancement of tumorigenesis and tumor progression through interaction with the host immune system (bottom)

The link between intratumor microbes and cancer development has been correlatively established and three primary mechanisms have been demonstrated as potential modes of action (Garrett, 2015; Dzutsev et al., 2017; Ramirez-Labrada et al., 2020): (1) direct facilitation of tumorigenesis via increasing mutagenesis, (2) regulation of oncogenes or oncogenic pathways, and (3) reduction or enhancement of tumor progression via modulation of host immune system (Fig. 1).

Many microbes have evolved to produce compounds that can cause damage to DNA, cell cycle arrest, and genetic instability. The presence of bacteria producing such compounds in what will become the tumor microenvironment could directly increase mutagenesis of the occupied tissue. Colibactin, produced via the *pks* locus in some *Escherichia coli* and other *Enterobacteriaceae*, exemplifies the potential of microbiota to directly promote oncogenesis (Nougayrede et al., 2006; Putze et al., 2009): it causes double stranded DNA damage and thereby facilitates tumorigenesis in colorectal cancer (Guerra et al., 2011; Garrett, 2015).

Aside from direct damage to DNA, commensal microbial products can engage with host oncogenic pathways. Upregulation or activation of pathways leading to carcinogenesis has been reported, notably including the Wnt/β-catenin signaling pathway (Garrett, 2015). Altered β-catenin signaling can promote transcription of oncogenes like *c-Myc* and *CyclinD-1* and advance carcinogenesis and tumor progression (Shang et al., 2017). Activation or modulation of β-catenin by local commensal microbiota has been reported in gastric and colorectal cancers (Sears, 2009; Kostic et al., 2012, 2013; Rubinstein et al., 2013; Abreu and Peek, 2014).

Immune modulation by commensal microbiota is well documented, as are its effects on carcinogenesis and cancer progression (Belkaid and Naik, 2013; Belkaid and Hand, 2014; Garrett, 2015; Dzutsev et al., 2017). The interaction between commensals and host immune system is well-balanced and homeostatic in a state of health. The host immune system is able to tolerate commensal microbial communities and respond appropriately to potentially harmful pathogens. However, perturbation of this balance and dysregulation of the microbiome can lead to the creation of microenvironments that promote tumor initiation and progression (Belkaid and Hand, 2014; Garrett, 2015). Many studies have shown that dysbiosis in local bacterial communities can cause a chronic, pro-inflammatory immune response, and thereby promote cancer growth. For example, this may occur through microbe activation of NF-κβ, a key regulator of cancer-associated inflammation (DiDonato et al., 2012; Elinav et al., 2013; Kostic et al., 2013; Garrett, 2015). Aside from inflammation, local microbes have been shown to modulate local immune surveillance by suppressing the antitumoral immune response. This type of immunosuppression has been observed in colon cancer patients, as well as in mouse models of lung cancer (Garrett, 2015; Gur et al., 2015; Le Noci et al., 2018), suggesting that the local commensal microbiota may be a potential target for cancer treatment, though further investigation is required.

## LUNG CANCER

As the mucosal site with the largest surface area in the body and a major interface with the external environment, the lung presents a unique opportunity for exposure to microbes and environmental challenges. Though traditionally thought to be sterile, the lung harbors a diverse array of microbes (Charlson et al., 2011; Erb-Downward et al., 2011). The myth of lung sterility stems from an inability to culture bacteria from lower respiratory tract samples (Baughman et al., 1987; Thorpe et al., 1987). However, with the advent of culture independent 16S rRNA sequencing technologies, diverse microbial populations have been discovered (Dickson et al., 2015; Yu et al., 2016). The lung microbiota is distinct from that of the gut and skin. In the healthy lung, *Prevotella*, *Streptococcus*, *Veillonella*, *Neisseria*, *Haemophilus*, and *Fusobacterium* are among the most abundant bacteria genera (Yu et al., 2016; Sommariva et al., 2020). Microbe prevalence is dictated by the immigration of new bacteria, mechanical and immune based elimination, and replicative success governed by local environmental conditions (Dickson et al., 2014; Sommariva et al., 2020). The lung microbiota has been shown to be in a state of dysregulation in pulmonary diseases like chronic obstructive pulmonary disease (COPD) and cystic fibrosis (Hilty et al., 2010; Erb-Downward et al., 2011; Dickson et al., 2013; O’Dwyer et al., 2016). Increasing evidence from both human and mouse studies have linked bacterial dysbiosis with lung cancer (Lee et al., 2016; Yu et al., 2016; Cameron et al., 2017; Liu et al., 2018; Ramirez-Labrada et al., 2020).

Among cancers, lung cancer is the leading cause of cancer related mortality and second most common cancer type, contributing to one quarter of all US cancer deaths (Siegel et al., 2019). An estimated 90% of all lung cancer cases are attributed to smoking, with tobacco smoke, air pollution, and other carcinogens all established risk factors, though exact mechanisms are not well understood (Gustafson et al., 2010). Although there are controversies (Greathouse et al., 2020), the intratumor and intracellular bacterial taxa of smokers show enrichment in degradation pathways for chemicals in cigarette smoke, pointing towards an association between intratumor microbiome and cancer etiology (Nejman et al., 2020). More generally, the lung commensal microbiota has been linked to cancer. For example, Yu et al. found a correlation between increased *Thermus* genus abundance and advanced stage cancer, as well as between *Legionella* and metastases. Additionally, a decrease in the alpha diversity of bacterial community in tumor tissues was reported in comparison to non-malignant lung tissues in lung cancer patients (Yu et al., 2016). Though methods and samples vary, other studies have recapitulated an enrichment of specific bacterial taxa and reduced diversity in association with lung cancer (Yan et al., 2015; Lee et al., 2016; Cameron et al., 2017; Greathouse et al., 2018; Tsay et al., 2018; Gomes et al., 2019). Differences between non-malignant and tumor tissue point towards the question: what is the role of tumor-associated microbiota, especially the intratumor microbiota, in tumorigenesis and progression of lung cancer?

The lung microbiota can directly affect the growth of lung cancer cells. Of the three main mechanisms we have presented for the role of local commensal microbes in carcinogenesis and cancer progression, regulation of local immune environment, and oncogenic pathways have been implicated in lung cancer. Dysregulation of lung microbial communities has been suggested to facilitate changes in oncogenic pathways, potentially through specific microbial components (Ramirez-Labrada et al., 2020). Findings by Greathouse et al. suggested an association between *TP53* and lung microbiota dysbiosis. The genus *Acidovorax* was found to be enriched in squamous cell carcinoma lung biopsy samples; this same taxa was found to be further enriched in lung biopsies of *TP53* mutated squamous cell carcinoma patients, though further mechanistic analysis remains to be pursued (Greathouse et al., 2018). Using airway brushings from diagnostic bronchoscopies, Tsay et al. found that patients with lung cancer had increased oral taxa, specifically *Streptococcus* and *Veillonella*, compared to controls. Increased prevalence of oral taxa was associated with PI3K and ERK upregulation. *In vitro* experiments exposing airway epithelial cells to *Veillonella*, *Prevotella*, and *Streptococcus* also resulted in ERK and PI3K pathway upregulation (Tsay et al., 2018). The PI3K pathway has been implicated as an early event in lung carcinogenesis (Gustafson et al., 2010), and therefore upregulation of this pathway by commensal microbiota dysbiosis facilitates carcinogenesis.

Lung microbiota has been shown to alter the immune microenvironment to promote tumor progression. Chronic inflammation has been closely linked to cancer; cytokines, chemokines, and other pro-inflammatory factors can facilitate tumor growth and spread (Garrett, 2015; Sommariva et al., 2020). The lung, due to its extensive exposure to the external environment, is a critical site of immune–microbiota interaction and exists in homeostasis maintained by lung resident immune cells (Pilette et al., 2001; Huffnagle et al., 2017; Lloyd and Marsland, 2017; Sommariva et al., 2020). Jin et al. demonstrated the importance of microbiota-immune crosstalk in promoting inflammation and the development of lung cancer in an autochthonous mouse model. Specifically, they found that certain bacterial families such as *Herbaspirillum* and *Sphingomonadaceae* were enriched in tumor-bearing lung tissues compared to healthy lungs, while other taxa including *Aggregatibacter* and *Lactobacillus* were enriched in healthy lungs. The increased local bacterial burden and altered composition of lung microbiota stimulated Myd88-dependent IL-1β and IL-23 production from myeloid cells. These cytokines induced the activation and proliferation of Vy6+Vδ1+γδ T cells, which produced IL-17, promoting inflammation and neutrophil infiltration. Additionally, these γδ T cells produced IL-22 and other effector molecules promoting tumor cell proliferation. Germfree (GF) mice or antibiotics-treated mice had significantly reduced lung tumor growth, demonstrating commensal bacteria significantly promoted lung cancer development (Jin et al., 2019). Similarly, Le Noci et al. utilized aerosolized antibiotics to demonstrate that decreased bacterial biomass was linked to an enhancement of antitumoral immune response via T cell and NK cell activation and reduction of immunosuppressive regulatory T cells. Additionally, the probiotic *Lactobacillus rhamnosus* was found to overcome immunosuppression and inhibit lung tumor implantation, and tumor metastases were reduced under both antibiotic and probiotic conditions (Le Noci et al., 2018). Altogether, these findings support the notion that local microbiota plays a key role in lung cancer development by modulating the local immune response and targeting tumor-associated microbiota presents a potential new avenue for lung cancer prevention and treatment.

## GI CANCER

### Colorectal cancer

The gastrointestinal tract harbors the vast majority of commensal microbiomes in the human body (Dzutsev et al., 2017), and bi-directional crosstalk between the host and the microbiome communities has emerged as a central aspect of both tumor progression and the therapeutic response to various types of GI cancers (Garrett, 2015; Dzutsev et al., 2017; Shalapour and Karin, 2020).

Numerous studies have established the role of commensal bacteria in colorectal cancer. Mechanistically, gut microbiota has been shown to promote mutagenesis by causing double-stranded DNA damage in host, or through activating oncogenic signaling like the Wnt/β-catenin pathway, or promoting inflammation by upregulating NF-kB signaling via increased engagement of pattern recognition receptors (Arthur et al., 2012; Kostic et al., 2013; Rubinstein et al., 2013). For example, *Fusobacterium* was first identified to be associated with colorectal carcinoma using unbiased genomic analyses (Castellarin et al., 2012; Kostic et al., 2012). Subsequent study further demonstrates the persistent colonization of *Fusobacterium nucleatum* and its associated anaerobes in colorectal tumors (Bullman et al., 2017). Bullman and colleagues, using both *in vitro* and *in vivo* human-derived colorectal cancer (CRC) xenograft models, showed that *F*. *nucleatum* promotes tumor cell proliferation or tumor growth (Bullman et al., 2017). A possible mechanism explaining this finding is the binding of *F*. *nucleatum* adhesin molecule to the cell surface motifs on cancer cells or immune cells, leading to the downstream oncogenic or immunosuppressive signaling (Garrett, 2019). Other commensal bacteria associated with colorectal cancer include enterotoxigenic *Bacteroides fragilis* (ETBF) and *Escherichia coli* expressing the genomic island polyketide synthase (pks+ *E*. *coli*) (Arthur et al., 2012; Dejea et al., 2018). It is discovered that the pks+ *E*. *coli*-derived colibactin alkylates DNA and produces DNA adducts, resulting in DNA damage in colonic epithelial cells (Wilson et al., 2019). Dejea et al. found that cocolonization by ETBF and pks+ *E*. *coli*, as shown by FISH and microbiology culture analysis, can accelerate the onset of colon cancer and increase mortality in mouse models of CRC (Dejea et al., 2018). In addition, tumor-coating ETBF has been shown to recruit other bacteria, as well as immune cells, to the tumor site and boosts IL-17-mediated inflammation (Dejea et al., 2018). Interestingly, a recent study discovered that mutant *p53* is switched from tumor-suppressive to oncogenic by the gut microbiota in the distal gut i.e., ileum and colon (Kadosh et al., 2020).

Aside from tumorigenesis, intratumor microbiota plays an important role in modulating the response to cancer therapies. For instance, *F*. *nucleatum* was found to be abundant in the CRC tumorous tissues in patients with recurrence post chemotherapy (Yu et al., 2017). Yu et al. showed that *F*. *nucleatum* promotes chemoresistance by activation autophagy pathway through downregulation of microRNAs (miR-18a and miR4802) (Yu et al., 2017). In another study that investigated the role of intratumor microbiota in CD47-based cancer immunotherapy, Shi et al. found that colonic *Bifidobacterium* accumulates in tumor sites and facilitates local anti-CD47 treatment via the STING pathway (Shi et al., 2020).

## OTHER GI CANCERS

Pancreatic cancer is one of the deadliest cancers and has a very poor prognosis, with a five-year survival rate of 8% (Siegel et al., 2018). The pancreas was historically considered to be sterile, yet emerging evidence demonstrates the presence of intratumor microbes and their impact on pancreatic cancer progression and therapeutic efficacy (Balachandran et al., 2017; Geller et al., 2017; Pushalkar et al., 2018; Aykut et al., 2019; Riquelme et al., 2019; Nejman et al., 2020). Geller and colleagues (Geller et al., 2017) incidentally found that 76% of the human pancreatic ductal adenocarcinoma (PDAC) samples were positive for bacteria, with *Gammaproteobacteria* being the dominant taxa. Bacteria cultures from 14 out of 15 (93%) fresh human PDAC tumors were able to confer resistance to chemotherapeutic drug gemcitabine on tested human colon cancer cell lines. Pushalkar et al. (Aykut et al., 2019) discovered an increased abundance of select bacteria (45% of which were *Proteobacteria*) in pancreatic tumors from genetically engineered mice models. It was demonstrated that these intratumor bacteria led to T cell anergy in a Toll-like receptor (TLR)-dependent manner, and transfer of selective or bulk fecal bacteria from PDAC-bearing mice, but not control mice, accelerated tumor progression. In addition to bacteria, intratumor fungi like *Malassezia globosa* in pancreatic cancer were also found to contribute to tumorigenesis, tumor growth, and gemcitabine resistance in a mannose-binding-lectin-dependent manner (Aykut et al., 2019). On the other hand, some intratumor microbiomes were reported to boost the anti-tumor immunity. For example, Riquelme and colleagues (Riquelme et al., 2019) found that the abundance of intratumor bacteria *Pseudoxanthomonas*, *Saccharopolyspora*, and *Streptomyces* spp. was highly predictive of long-term survival in pancreatic cancer patients, and an overall more diverse composition of the tumor microbiome was observed in long-term survivors; the more diverse microbiome in the tumor contributes to the anti-tumor immune response by favoring recruitment and activation of CD8^+^ T cells. Furthermore, Balachandran et al. (2017) discovered that the enhancement of immune infiltrates was associated with the presence of intratumoral neoantigen MUC16 in long-term survivors. The authors speculated that intratumor microbiomes may enhance immune identification of relevant neoantigens (Balachandran et al., 2017).

In esophageal cancer, the presence of *F*. *nucleatum* in esophageal cancer tissues was correlated with a poor prognosis (Yamamura et al., 2016). Mechanistically, Yamamura et al. (2016) suggested that *F*. *nucleatum* contributed to tumor infiltration by Treg lymphocytes in a chemokine (especially CCL20)-dependent fashion, thereby promoting aggressive tumor behaviors.

As commensal microbiota resides in the GI tract at the steady state, an important question is posed as the origin of tumor-associated bacteria. Consistent with the hypothesis that constant interactions exist between the pancreas, liver, and intestine, the development of pancreatic and liver cancer has been associated with the dysregulation and mislocation of gut microbiota (Bullman et al., 2017; Geller et al., 2017; Pushalkar et al., 2018; Vitiello et al., 2019). Geller et al. (2017) suggested that PDAC-associated bacteria could be sourced from the gastrointestinal tract in a retrograde manner. Pushalkar et al. (2018) showed evidence of bacteria migration from the gut to the pancreas, as well as a time-dependent association between gut dysbiosis and *Kras* activation in PDAC. Gut dysbiosis can also directly promote oncogenic signaling in the pancreas (Vitiello et al., 2019). Commensal microbiome obtained from long-term PDAC survivors was highly capable of enhancing immune infiltration and anti-tumor immunity (Riquelme et al., 2019). As in liver cancer, Bullman et al. (2017) found that the relative abundance of *Fusobacterium* was significantly increased in colorectal cancer-derived liver metastases in comparison to primary liver hepatocellular carcinoma. The crosstalk between these organs points to a need to elucidate the origin of the observed intratumor microbiomes in pancreatic and liver cancer, and the relative contribution of microbiome in the distal intestine or in the local cancerous tissue to cancer-associated immune response.

## SKIN CANCER

The human skin is home to thriving populations of diverse microbes, with an estimated one million bacteria and hundreds of different species per square centimeter (Chen and Tsao, 2013). As in other body sites, changes in the composition of the skin microbiome are associated with cancer. In a study of the microbiota of normal versus melanotic pig skin, the melanoma samples were found to be enriched in *Fusobacterium* and *Trueperella* genera (Mrázek et al., 2019). In a cell culture study, a strain of skin commensal microbes, *Staphylococcus epidermidis*, had a protective effect against skin cancer. These strains of *Staphylococcus epidermidis* produce 6-N-hydroxyaminopurine, an inhibitor of DNA polymerase activity, which stops tumor line proliferation in culture (Nakatsuji et al., 2018). Investigation of the relationship between host immune system, local microbial communities, and cancer has also begun in the context of skin cancer. Hoste et al. utilized a wound-induced skin cancer mouse model to interrogate the mechanisms through which the skin microbiota promotes inflammation and tumorigenesis. In the presence of skin microbes, elimination of several innate immune sensors, including TLR-5, protects from tumorigenesis, while inflammation correlates with tumor incidence. Treatment with antibiotics inhibits tumor formation in a TLR-5 dependent manner (Hoste et al., 2015). A better understanding about the role of the skin microbiome in skin cancer is necessary, as it could potentially provide further insight into the different roles tissue-specific microbiota in cancer initiation, progression, and potentially treatment.

## OTHER CANCERS

In addition to the major mucosal organs described above, the vaginal microbiome has also been associated with cervical cancer (Shannon et al., 2017; Norenhag et al., 2020). Aside from tumors that arise from the mucosal sites that host tissue-resident commensals, it is interesting to note that intratumor bacteria has also been identified in other types of cancer, including breast cancer, ovarian cancer, bone cancer, and glioblastoma multiforme (GBM) (Hieken et al., 2016; Urbaniak et al., 2016; Banerjee et al., 2018; Nejman et al., 2020). However, there is very limited experimental evidence for association of distinct microbes with these types of cancer. A recent landmark study by Nejman et al. (2020) showed the presence of intratumor bacteria in ovarian and bone cancers, as well as in GBM. Distinct microbial compositions and metabolic functions encoded by intratumor bacteria were identified across different types of cancer (Nejman et al., 2020). Moreover, different bacterial signatures were associated with subtypes of breast cancer, in agreement with findings from previous articles (Urbaniak et al., 2016; Banerjee et al., 2018). In addition, it was discovered that breast tumor-associated microbiomes had the highest level of diversity and abundance among all tumor types tested (Nejman et al., 2020). Importantly, this study confirmed the presence of metabolically active bacteria using *ex vivo* bacterial isolation from fresh breast tissue and fluorescently labeled *D*-alanine.

## CONCLUSION AND DISCUSSION

The microbiota is gaining increasing attention as a key player in the tumor microenvironment that modulates tumor progression and influences cancer prognosis. The microbial communities of different body sites play a role in tumorigenesis and tumor progression of their respective cancers. In many cases, direct changes in mutagenesis, regulation of oncogenic pathways, and modulation of the immune system can provide potential explanations for the link between local microbes and cancer (Garrett, 2015; Dzutsev et al., 2017; Ramirez-Labrada et al., 2020). Notably, the commensal microbiota exerts an effect upon the immune microenvironment, frequently promoting inflammation or dampening anti-tumor immunity. Crosstalk between the host immune system and local microbes affects cancer growth and spread (Belkaid and Hand, 2014; Garrett, 2015; Ramirez-Labrada et al., 2020).

Despite our growing knowledge of microbiota in cancer and cancer treatments, the role of tumor-associated bacteria requires further investigation. Untangling the complex relationships between microbes, the tumor microenvironment, and cancer cells could provide valuable insight into potential cancer treatments and the way existing cancer treatments might perform in individual cancer patients. While the gut microbiota has been extensively studied in the context of carcinogenesis and tumor response to immunotherapy (Gopalakrishnan et al., 2018; Matson et al., 2018; Routy et al., 2018), the microbiota of the lung and other less well studied body sites requires better understanding.

Questions remain to be addressed in this growing field. For example, do tumor-associated microbes originate from the local expansion of normal tissue-resident commensals, or are they recruited from elsewhere as tumors progress? If tumor-associated microbial communities represent a shift from homeostasis, how does this shift occur spatially and temporally? Do the mechanisms of commensal microbe action differ between distinct types of cancer, and how do they differ? Other than microbial products, what is the contribution of the microbial metabolite within and around the tumor? One of the key challenges in the field is to selectively manipulate the microbiota of a specific body or tumor site without affecting the gut microbiota and the relatively low microbial presence in organs like the lung (Jin et al., 2019). Overall, future study of the intratumor microbiota is needed to explore its role in cancer and to find new therapeutic strategies for cancer treatment, including targeted and individualized treatments to maximize the efficacy of anti-tumor therapies.
